# 3,3-Disubstituted 3,4-Dihydro-1,2,4-benzotriazines: Chemistry, Biological Activity, and Affinity to Sigma Receptors

**DOI:** 10.3390/molecules29010132

**Published:** 2023-12-25

**Authors:** Fabio Sparatore, Anna Sparatore

**Affiliations:** 1 Department of Pharmacy, University of Genova, 16132 Genova, Italy; 2 Department of Pharmaceutical Sciences (DISFARM), University of Milano, 20133 Milano, Italy; anna.sparatore@unimi.it

**Keywords:** 3,4-dihydro-1,2,4-benzotriazines, 2-nitro-phenylhydrazone reduction, sigma receptor ligands, antihypertensive activity, anti-inflammatory activity, diuretic activity, antiproliferative activity

## Abstract

By reducing the 2-nitrophenylhydrazone of cyclohexanone with sodium dithionite, an unexpected yellow compound was obtained instead of the corresponding colorless amino derivative. Many years later, the structure of this compound, namely, cyclohexane-3-spiro-3,4-dihydro-1,2,4-benzotriazine, was demonstrated. From that time, the reduction of 2-nitrophenylhydrazones of different kinds of ketones, followed by air oxidation of the initially formed amino compounds, has represented a general way to synthesize a variety of 3,3-disubstituted 3,4-dihydro-1,2,4-benzotriazines. Many derivatives have been obtained so far by a single research group, and most of them have demonstrated interesting pharmacological activities, mainly antihypertensive, anti-inflammatory, and diuretic effects and other activities with lower diffusion. Moreover, 3,3-disubstituted 3,4-dihydro-1,2,4-benzotriazines represent a novel class of ligands for sigma receptors, with nanomolar affinity to the σ_1_ subtype. This property might promote the development of agents for cardiovascular, neurodegenerative, and proliferative pathologies. The present commentary, by collecting compounds and biological results obtained so far, intends to celebrate the centennial of the discovery of the first member of this class of compounds and to promote further investigation in the field.

## 1. Introduction

One century ago, W. H. Perkin Jr. and G. C. Riley [1], by reducing the 2-nitrophenylhydrazone of cyclohexanone **1** with sodium dithionite, expected to obtain the corresponding amino derivative to be, successively, converted to 8-amino-1,2,3,4-tetrahydrocarbazole. Instead, they obtained a bright yellow compound not susceptible to Fischer indole cyclization and to which was assigned the structure **2** of a derivative of the benzotriazepine ring, at that time unknown.

**Scheme 1:**
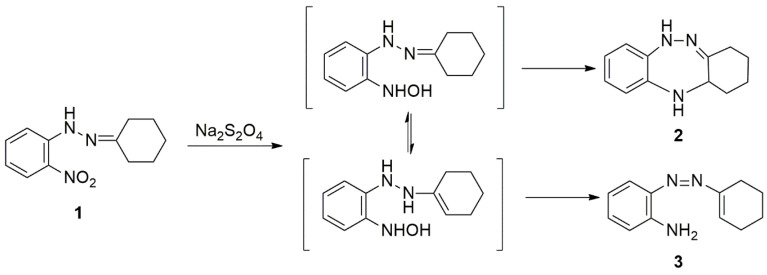
Reduction of cyclohexanone 2-nitrophenylidrazone: initially supposed pathway.

In 1955, during an investigation on benzotriazepine derivatives of potential pharmacological interest, F. Sparatore [2] observed that the Perkin and Riley compound undergoes a prompt diazotization reaction, thus postulating the presence in the molecule of a primary amino group and suggesting for it the open structure **3** of a 2-aminophenylazocyclohexene (Scheme 1).

**Scheme 2:**
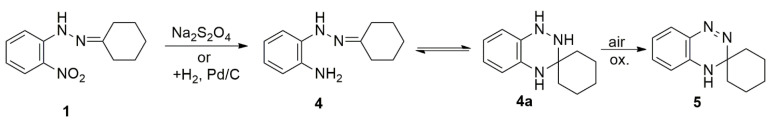
Reduction of cyclohexanone 2-nitrophenylhydrazone: formation of cyclohexane-spiro-3,4-dihydro-1,2,4-benzotriazine.

Later, after a more exhaustive examination, F. Sparatore and Cerri [3,4] established, for the relevant compound, the structure **5** of cyclohexane-3-spiro-3,4-dihydro-1,2,4-benzotriazine that is formed in two steps; the initial reduction of the nitro to amino group **4** and subsequent air oxidation of the 1,2,3,4-tetrahydrobenzotriazine **4a**, formed by the nucleophilic attack of the amino group on the hydrazone carbon (Scheme 2):

Since then, many 3,3-disubstituted 3,4-dihydro-1,2,4-benzotriazines have been synthesized by a single Italian research group and found to be endowed with a variety of bio-pharmacological activities.

The present review, by collecting compounds and biological results so far obtained, is intended to celebrate the centennial of the discovery of the first member of this peculiar class of compounds and to promote further research in this field.

## 2. Formation of 3,4-Dihydro-(1,2,4)-benzotriazine Derivatives

The reaction sequence leading to compound **5** has been shown to have a general application, giving positive results in the presence of the most different substituents on the benzene ring or even by exchanging the last with a pyridine nucleus (starting from 3-nitro-pyrid-2/4-yl-hydrazine) and maybe with other heteroaromatic rings.

On the other hand, all kinds of ketones (aliphatic, alicyclic, and aryl aliphatic) are suitable for the reaction, provided that bulky groups are not close to the carbonyl function [5]. Starting from aryl alkyl ketones, the formation of dihydrobenzotriazine derivatives can be rather difficult, but in these cases, the electronic effects of the aromatic ring are to be added to the only moderate steric hindrance. In fact, starting from acetophenone, the yield is very low (2.6%), while from methyl cyclopentyl ketone (of comparable bulkiness), the yield is 61% [5].

During the air oxidation of 2-aminophenylhydrazones, the dihydrobenzotriazine derivatives are always formed together with a complex mixture of alteration products, whose composition has not been studied thoroughly. However, a certain amount of the starting ketone and of aniline is always present, and their quantities increase as the yield of dihydrobenzotriazine derivatives decreases. These collateral products are, most probably, formed in an alternative way, but it is not excluded that, at least in part, they are formed by further oxidative breakdown of the dihydrobenzotriazine derivatives themselves. Generally, 3,3-disubstituted dihydrobenzotriazine derivatives are quite stable; a sample of compound **5** stored at room temperature is still unchanged after about 70 years from its preparation. However, even small quantities of impurities (particularly for some compounds) may foster the alteration in a progressively accelerated way.

**Scheme 3:**
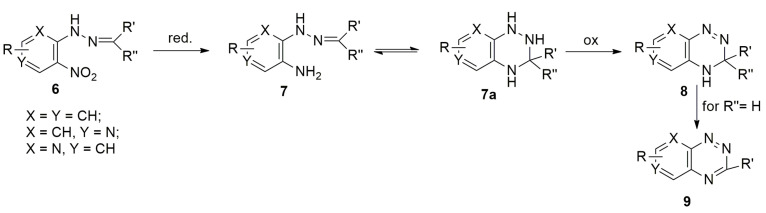
General pathway of the formation of 3,4-dihydro-1,2,4-benzotriazines and analogs.

Aliphatic and aromatic aldehydes may also be used [6], but of course the 3-monosubstituted 3,4-dihydrobenzotriazines are readily oxidized to the aromatic benzotriazines **9** (Scheme 3). Therefore, the air oxidation of aldehyde 2-aminophenylhydrazones represents an alternative method for the synthesis of 3-substituted 1,2,4-benzotriazines, which is competitive with the well-established methods of their preparation [7].

Reduction of 2-nitrophenylhydrazones may be accomplished in many ways, but the best (quantitative) results are obtained by catalytic hydrogenation on Pd/C, thus avoiding any collateral effect of the reactants (see Chemical properties section). The oxidation of tetrahydrobenzotriazines is accomplished by bubbling air (rarely O_2_) into their ethanolic solution. Excess activated MnO_2_ is used for the oxidation of tetrahydropyridotriazines [see further].

If the addition of the amino group to the hydrazone double bond is precluded, the corresponding open form of 2-aminophenylhydrazone **7** may be isolated as colorless rather unstable crystals, as seen in the case of the camphor derivative [2].

**Scheme 4:**
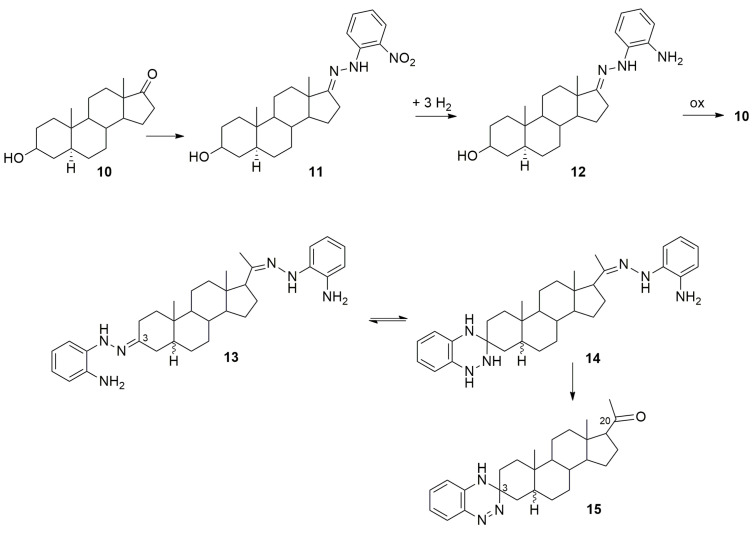
Effects of steric hindrance on heterocyclic formation.

In the case of 5α-epi-androsterone **10**, because of the overwhelming steric hindrance around C-17, it is even difficult to obtain 2-nitrophenylhydrazone; however, once formed, the reduction and subsequent oxidation of the amino derivative yields the starting ketone, with degradation of the aromatic moiety [8]. Thus, in the case of compounds with two (or more) isolated carbonyl groups affected, respectively, by different degrees of steric hindrance, it would be possible to build up the dihydrobenzotriazine ring only on the position of the less hindered carbonyl group. For instance, starting from 5α/5β-pregnane-3,20-dione bis 2-aminophenylhydrazone **13** [8], the dihydrobenzotriazine nucleus will be formed only on position 3, while the carbonyl function will be restored at position 20 through the breakdown of the 2-aminophenylhydrazone moiety (Scheme 4).

**Scheme 5:**
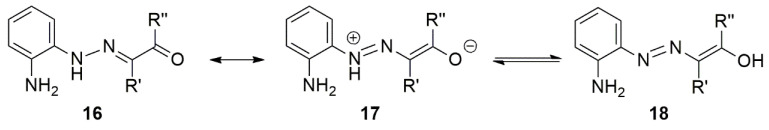
Stability of α-diketone 2-aminophenylhydrazones.

It is worth noting that the formation of the dihydrobenzotriazine ring is also precluded in the case of mono 2-aminophenylhydrazone of α-dicarbonyl compounds because of stabilization of the open form by resonance and/or the formation of azo-enols (Scheme 5) [9].

**Scheme 6:**
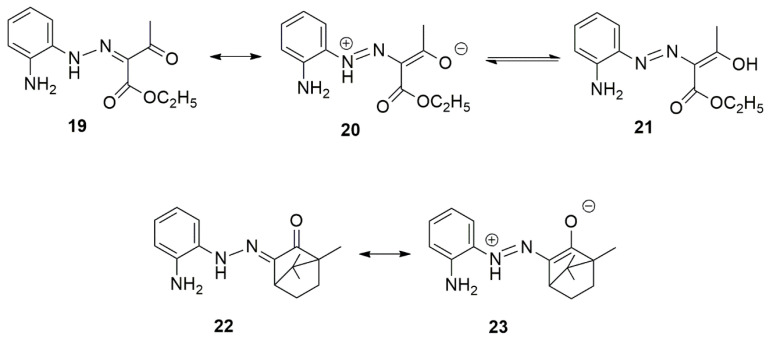
Examples of stable α-diketone 2-aminophenylhydrazones.

Indeed, by reducing the ethyl 2-(2-nitrophenylhydrazone)-3-oxo-butyrate [9] or the camphorquinone α-mono(2-nitrophenylhydrazone) [10], two very stable amino compounds **19** and **22** are obtained (Scheme 6). In the first case, two interconvertible crystalline forms are isolated, one red and low melting (from petrol ether) and the other yellow and high melting (from ethanol), which might represent the carbonylic **19** and the enolic form **21**, respectively, not excluding, however, a case of simple crystal dimorphism.

The enhanced chemical stability of these mono 2-aminophenylhydrazones (**19** and **22**) not only precludes the cyclization to dihydrobenzotriazines but also allows other transformations only occasionally and under energic conditions [10].

Starting from ketones bearing two different groups, R’ and R”, inseparable enantiomeric forms are formed.

## 3. Chemical Properties of 3,4-Dihydro-1,2,4-benzotriazines

**Scheme 7:**
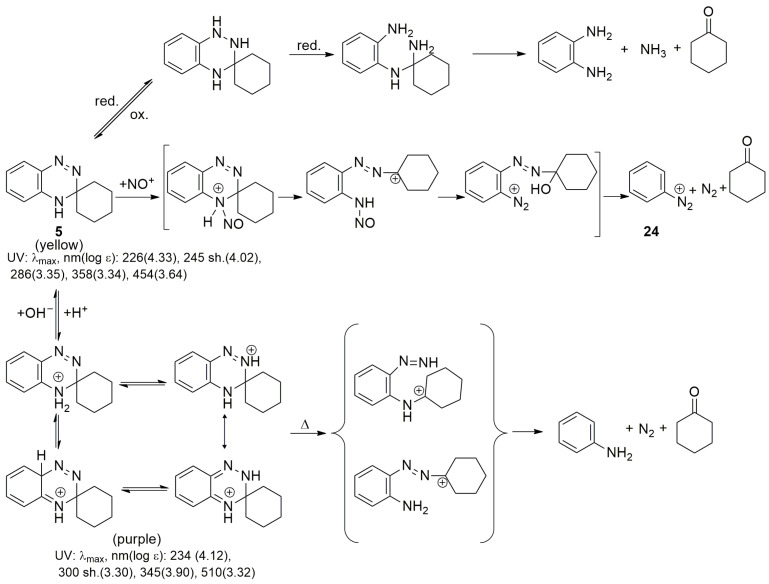
Main reactions of 3,4-dihydro-1,2,4-benzotriazine **5**.

As already stated, 3,3-disubstituted 3,4-dihydro-1,2,4-benzotriazines are very stable in the pure solid state. They are characterized by a bright yellow color that in acid medium turns to intense red purple related to a number of tautomeric and resonating ionic forms. At room temperature, they remain unchanged for a few days in 2N hydrochloric acid; however, with longer incubations or warming, they are decomposed, forming aniline (or substituted aniline) and the starting ketone. Under catalytic (Pd/C) hydrogenation, they absorb one single mole of hydrogen, losing their color, but the compounds so obtained give rise to the starting yellow compounds when exposed to air. It should be noted that the tetrahydrobenzotriazines (cyclic forms of 2-aminophenylhydrazones) are cleaved easily by acids to give the starting ketones. Reduction with zinc and acetic acid gives rise to ortho-phenylenediamine, ammonia, and ketone. Very importantly, nitrous acid, through its tiny nitrosonium ion, is able to attack the hindered NH group, giving rise to complete breakdown of the molecule and final formation of ketone and phenyl diazonium ion **24** (Scheme 7) [4].

**Scheme 8:**
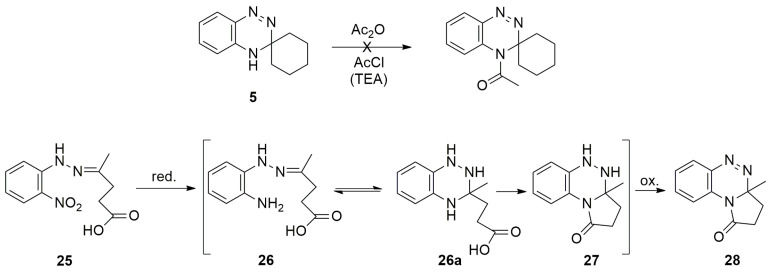
Intramolecular acylation of the NH group.

**Scheme 9:**
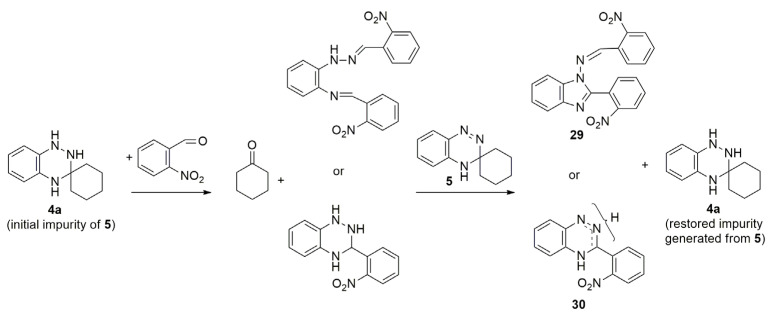
Condensation of impure compound **5** with 2-nitrobenzaldehyde.

Because of steric hindrance, the NH group is not acylated, neither by acetic anhydride nor by acetyl chloride (and TEA). However, the sequence of catalytic reduction and air oxidation of 2-nitrophenylhydrazone of levulinic acid **25** gives rise to the tricyclic acylated compound **28** [11]. It is supposed that the intramolecular acylation occurs at the stage of the more flexible tetrahydrobenzotriazine (Scheme 8):

Pure cyclohexane-3-spiro-3,4-dihydro-1,2,4-benzotriazine **5** does not react with 2-nitrobenzaldehyde; but if compound **5** is impure with even a very small quantity of the corresponding cyclohexanone 2-aminophenylhydrazone (**4**-**4a**), a substantial amount of a red condensation compound is isolated after long heating [2]. This fact may be explained through a chain reaction involving the spiro compound **5** as an acceptor of hydrogen, thus regenerating the tetrahydro compound that is progressively condensed with the 2-nitro-benzaldehyde (Scheme 9).

## 4. Pharmacological Activities

A large number of 3,3-disubstituted derivatives of 3,4-dihydro-1,2,4-benzotriazine have been prepared, bearing at position 3 moieties of various complexity, eventually containing chemical functions of neutral, acidic, or basic nature, which could, of course, influence chemical and biological characteristics.

**Figure 1:**
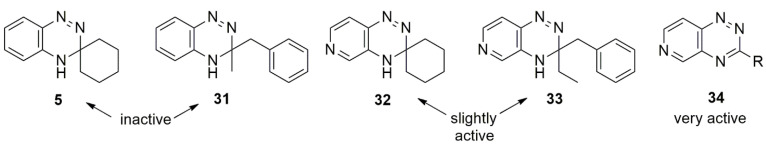
Comparison between dihydrobenzotriazine and dihydropyridotriazine.

To explore the biological activity inherent to this novel type of heterocycle, two compounds bearing at position 3 relatively inert groups (as the spiro-derivative **5** and the 3-benzyl-3-methyl-3,4-dihydro-1,2,4-benzotriazine **31**, Figure 1) were subjected to an extensive screening, with in vitro and in vivo tests, in order to reveal any CNS stimulation or depression or analgesic, anti-inflammatory, antihypertensive, antiarrhythmic, or antimicrobial activities. Compounds were administered p. os at a dose of up to 300 mg/kg for mice and up to 150 mg/kg for rats; to investigate some activity, compounds were injected s.c. or i.p. at a dose from 50 to 100 mg/kg. The two compounds turned out to be inactive over the whole explored fields. This result could be related to not only a rather low activity-toxicity of the novel heterocycle but also unfavorable pharmacokinetics due to the high lipophilicity of the chosen compounds [5].

Indeed, functionalized compounds display a variety of biological activities. Depending on the nature of the moiety introduced at position 3 and/or of the substituents decorating the benzene nucleus, a single activity or, more frequently, a cluster of different activities can be observed, as has been documented for the fully aromatic 1,2,4-benzotriazine derivatives [8,12,13].

The nature of substituents at position 3 may modulate the hydrophilic-lipophilic balance of the molecule and, hence, its pharmacokinetics as well as the binding capability versus receptor sites. Even more, the substituents of the benzene ring may influence the reactivity of the azo group versus the biological redox systems (particularly the azoreductases [14,15] of the intestinal bacteria or of the liver microsomes) and the possibility of molecular breakdown with amine and ketone release. Also, the isosteric replacement of the benzene nucleus with a pyridine ring can have important effects on the biological activity.

Thus, the more hydrophilic 3,3-disubstituted 3,4-dihydropyrido [3,4-e]1,2,4-triazine derivatives **32** and **33**, isosteric to the inactive dihydrobenzotriazine **5** and **31**, are endowed with moderate in vitro antifungal activity (various *Candida* and *Trichophyton strains*) [16] (Figure 1).

Interestingly, 3-substituted pyrido [3,4-e]triazines **34** [16] are even more potent as antifungal agents, indicating that the activity is due to the whole heterocycle molecule and not to compounds deriving from ring splitting, which could be possible for **32** and **33** but not for the fully aromatic **34**.

**Figure 2:**
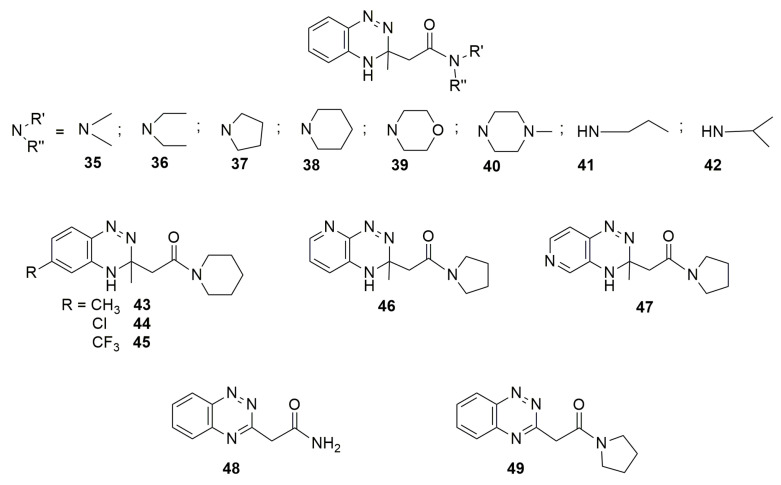
N-Substituted 3,4-dihydro-3-methyl-benzo-1,2,4-triazin-3-yl-acetamides and 3,4-dihydro-3-methyl-pyrido [3,2-e]/[3,4-e]-1,2,4-triazin-3-yl-acetamides.

On the basis of the high anti-inflammatory activity displayed by a number of amido derivatives of aryl/hetero-aryl alkanoic acids, particularly 1,2,4-benzotriazin-3-yl acetic acid [17,18], a group of thirteen amido derivatives of 3,4-dihydro-3-methyl-1,2,4-benzotriazin-3-yl/pyrido [3,2-e]/[3,4-e]triazin-3-yl acetic acid (**35**–**47**, Figure 2) were tested as inhibitors of carrageenan-induced rat hind paw edema and screened for several other activities [19]. Anti-inflammatory, antihypertensive, and diuretic activities were the strongest activities that were observed, besides minor negligible activities on the CNS.

Indeed, high and long-lasting levels of anti-inflammatory activity have been observed for eight compounds (**35**, **36**, **38**, **39**, **43**, **44**, **46** and **47**), with up to 80% inhibition after 4 h from oral administration of a dose of 25 mg/kg of morpholide **39**. Thus, in comparison to even the best of the inspiring fully aromatic analogs (as **48** and **49**, administered i.p. at a dose of 40–50 mg/kg), the tested 3,3-disubstituted amido compounds clearly exhibit superior potency and action duration. The observed improvement in the activity could be related to a peculiar out-of-plane disposition that the acetamido group is forced to assume with respect to the bicyclic system. Regrettably, no attempts have been made to separate enantiomers to verify the possible selective activity of the S(+) enantiomer as it is known for other anti-inflammatory agents.

Compounds **38** and **44** also displayed antihypertensive activity against DOCA-induced hypertension in rats. At the dose of 5 mg/kg i.p., this activity was comparable with, or quite superior to that of, guanabenz at the same dose (21% and 29%, respectively, versus 21.9% for guanabenz).

Diuretic activity was expressed by all compounds, with the only exception being compound **38**; very high levels of activity were noted for the secondary amines **41** and **42**, and these levels were comparable or superior (up to +110% of urine volume) to that produced by muzolimine. Noteworthy is the duration of diuretic effect, namely, that in some cases (**37**, **41** and **42**), the effect continues to increase even after 6 h. Interestingly, the most potent compounds as diuretics did not affect edema reduction or antihypertensive activity; therefore, they are not related to hypovolemia.

**Figure 3:**
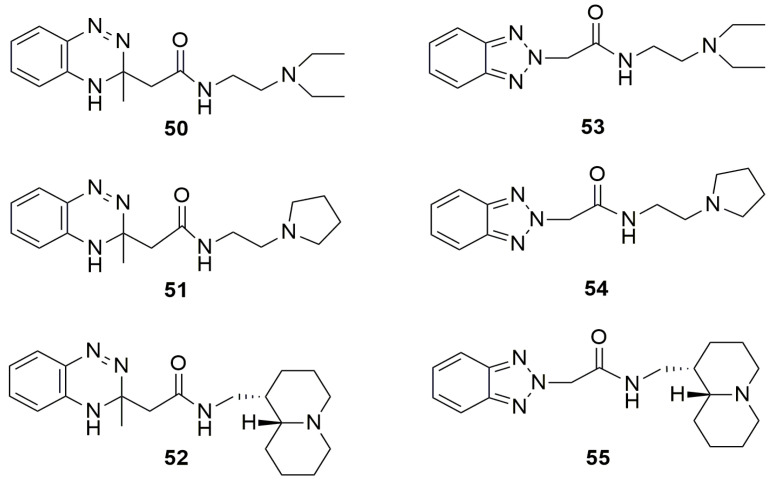
3,4-Dihydrobenzotriazinyl acetamides and benzotriazolyl acetamides as local anesthetics and antiarrhythmics.

Strictly related to the above derivatives of 3,4-dihydro-3-methyl-1,2,4-benzotriazin-3-yl-acetic acid are the three basic compounds **50**–**52** [20] that were designed and synthesized for comparison to analogous benzotriazole derivatives **53**–**55** (Figure 3) endowed with potent local anesthetic and antiarrhythmic activities [21,22,23]. The prepared compounds have not yet been tested.

**Figure 4:**
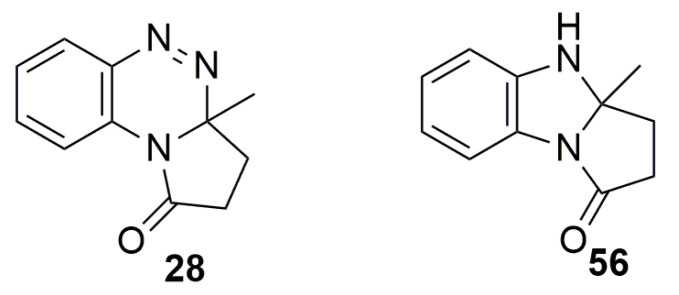
Structural analogy between anticonvulsant compounds.

To prepare 3,4-dihydro-3-methyl-1,2,4-benzotriazin-3-yl-propionic acid, the usual sequence of catalytic reduction and air oxidation is applied to the 2-nitrophenylhydrazone of levulinic acid. However, instead of the expected acid, the tricyclic lactam **28** [11] is obtained (see Scheme 8). The structure of this compound is similar to that of 3a-methyl-2,3,3a,4-tetrahydro-1*H*-pyrrolo [1,2-a]benzimidazol-1-one **56** [24] (Figure 4), which is endowed with anticonvulsant activity, comparable to that of diphenylhydantoin, against maximal electroshock seizure (MES) in rats (ED_50_, i.p. 35.5 and 55.5 µmol/kg, respectively).

In 1999, lactam **28** was included in the “Anticonvulsant Screening Project” of the National Institute of Neurological Disorders and Stroke of the NIH of Bethesda, MD, USA (as #298032), displaying an i.p. ED_50_ 149 µmol/kg (30 mg/kg) in MES tests, without any sign of toxicity up to 240 mg/kg. However, it was inactive against metrazol-induced seizures in mice and not further investigated (personal communication by dr. J. P. Stables).

**Figure 5:**
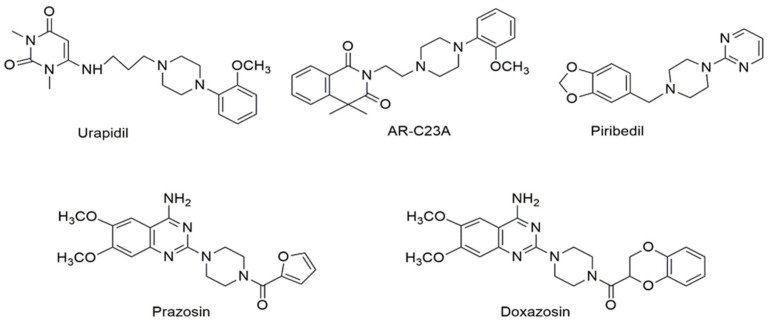
Structure of piperazine-derived antihypertensive drugs.

Valuable antihypertensive activity is displayed by compounds **38** and **44**,and its improvement was attempted through the hybridization of the dihydrobenzotriazine moiety with aryl-, heteroaryl, or acyl-piperazine scaffolds, which are present in well-known antihypertensive or vasodilator drugs (as prazosin, doxazosin, piribedil, urapidil, AR-C239 etc. (Figure 5)).

**Figure 6:**
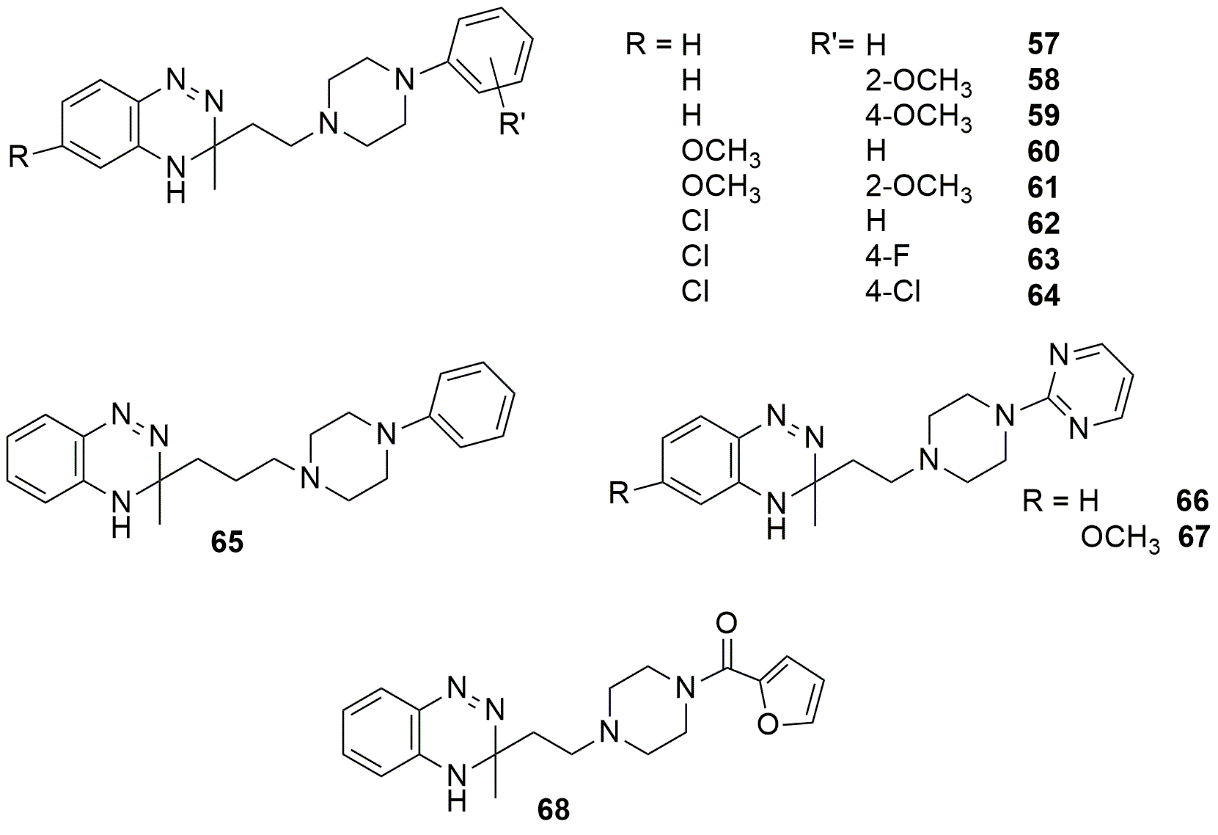
Structures of 3-[2-(1-aryl-piperazin-4-yl)ethyl]-3,4-dihydro-3-methyl-6-R-1,2,4-benzotriazines.

Indeed, piperazinyl derivatives can affect simultaneously or alternatively 5-HT_1A_, DA, and α_1_-adrenergic receptors depending on the nature of the substituents on the two nitrogen atoms [25]. Thus, the following compounds **57**–**68** (Figure 6) were prepared [26,27].

Only seven compounds, out of twelve, were tested for the sought-after antihypertensive activity in spontaneously hypertensive rats (SHRs). Among the tested compounds, only the furoyl derivative **68** was inactive, while all the others displayed high activity at 100 mg/kg p. os, although compounds **58**, **66**, and **67** displayed some toxicity. The best compounds were **61** and **67**, which at a dose of 30 mg/kg were comparable to nifedipine at 5 mg/kg.

The antihypertensive activity cannot be related to a single mechanism but seems to be the result of the addition of several minor effects observed in in vitro tests (as α_1_-adrenoreceptor and calcium antagonism, angiotensin inhibition, methacholine antagonism, etc.).

The structurally simplest compound **57** was subjected to a wide pharmacological screening that disclosed (a) inhibition of arachidonate-induced platelet aggregation (at 10 µg/mL conc.) and a consequent increase in bleeding time in mice; (b) relaxant activity on isolated zig-zag cut guinea pig trachea (at a conc. from 30 to 3 µg/mL), possibly related to the inhibition of cyclooxygenase (see preceding test) and not to β-adreno receptor agonism, being not antagonized by propranolol; (c) analgesic activity as evidenced by inhibition of phenyl quinone-induced writhing and prolongation of the time to elicit a pain response (tail flick test) in mice at 30 mg/kg p. os and i.p., respectively; and (d) glimpses of hypocholesterolemic activity (**57**; **62**) [26,27].

To improve the observed activities (anti-inflammatory, analgesic, antihypertensive, diuretic, anticonvulsant, and neuro-depressant) and to eventually disclose other kinds of activity, a number of novel 3,4-dihydro-1,2,4-benzotriazine derivatives were synthesized, where the two substituents of position 3 formed a basic cycle of varied dimensions and complexity.

**Figure 7:**
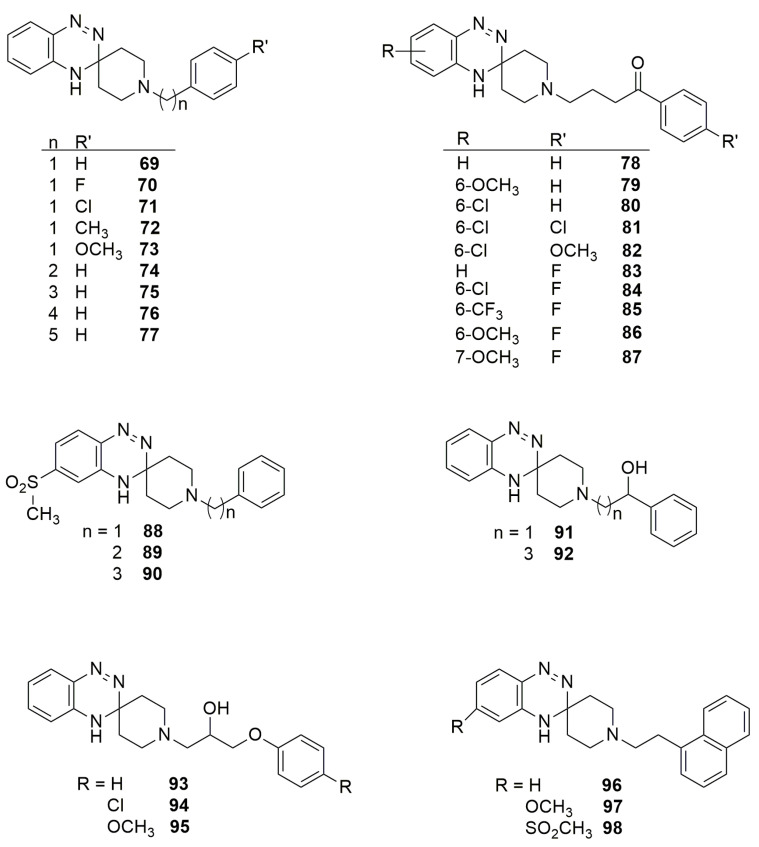
Structure of spiro [3,4-dihydro-6/7-R-1,2,4-benzotriazine-3,4′-(1-substituted)piperidines].

A first group of compounds is represented by the N-arylalkyl derivatives of spiro (3,4-dihydro-1,2,4-benzotriazine)-3,4′-piperidine **69**–**98** (Figure 7) [28].

The 30 prepared compounds could be allotted to several subsets, according to the kind of aryl-aliphatic chain, to which corresponded very diversified biological activities.

**Figure 8:**
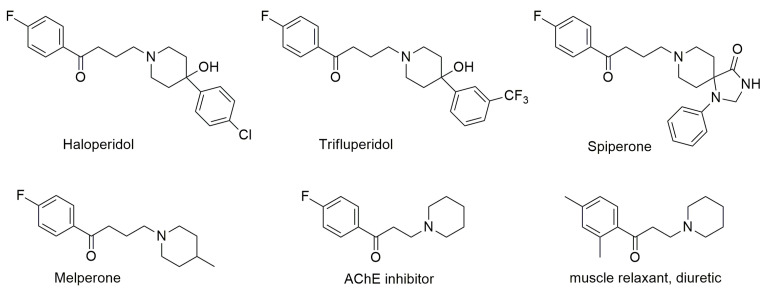
Structures of butyrophenone- and propiophenone-derived drugs.

A particular importance was attributed to compounds **83**–**87** [28], which with their N-(4-fluorobenzoyl)propyl side chain, resemble the well-known fluorobutyrophenone antipsychotic drugs, such as haloperidol, trifluperidol, and the very potent spiperone. It is worth noting that butyrophenone derivatives bearing simple substituents at position 4 of the piperidine ring, besides reduced antipsychotic activity, display high antiarrhythmic activity (melperone, [29]). Moreover, the propiophenone analogs, while losing the antipsychotic action, exhibit several other activities, such as acetylcholinesterase (AChE) inhibitory [30], central muscle relaxant, and diuretic effects [31] (Figure 8).

Consistent with their structural analogy to the butyrophenone drugs, compounds **83**–**86** display some behaviors related to depression in the Irwin test and/or in the apomorphine-induced climbing test, but affinity to the D_2_ receptor was rather low compared to that observed for **83** and **84** in the [^3^H]-spiperone displacement test in rat striate (Ki = 164 nM, compared to Ki = 8.7 nM for l-sulpiride, a classic ligand for the D_2_ receptor).

Compounds **83**–**86** showed high analgesic activity in the phenylquinone-induced writhing test and in the formalin algesia test, but only **83** and **84** were active in the tail flick test, indicative of central antinociceptive action (51%-time elongation at a dose of 3 mg/kg i.p. versus 50% for codeine at 20 mg/kg i.p.). On the other hand, only the 6-methoxy derivative **86**, at 100 mg/kg p. os, was able to reduce by 35% carrageenan-induced rat hind paw edema. This activity is comparable to that exerted by phenylbutazone at a dose of 50 mg/kg p. os., but, differently from this, compound **86** did not exhibit any sign of gastric irritation at the tested dose. The same compound did not inhibit the platelet aggregation induced by arachidonic acid (AA); therefore, no inhibition of cyclooxygenase should be involved in the edema reduction, which could be related to the antihistamine and antiserotonin activity observed in in vitro tests and/or the antihypertensive activity (see further). The inhibition of AA-induced platelet aggregation was seen only with a high concentration (100 µg/mL) of the 6-chloro derivative **84**. Analgesic activity for this set of compounds, particularly **83** and **86**, could be related to affinity to sigma-1 receptor (see further).

Importantly, a remarkable and long-lasting reduction in blood pressure was produced in spontaneously hypertensive rats (SHRs) by compounds **79**, **85**, and **86** at a dose of 100 mg/kg p. os [28]. The observed level of activity was comparable, or even superior, to that exerted by α-methyl-DOPA and nifedipine at their effective doses of 50 and 5 mg/kg, respectively. Only minimal activity was displayed by compounds **83** and **84**, while **87** was completely inactive. It is worth noting that there was a strong enhancing effect of the introduction of a methoxy group at position 6 (compare **86** with **83**), while its shift at position 7 (compound **87**) abolished completely the antihypertensive action. As it was observed for the aryl-piperazino-ethyl derivatives **61** and **67**, also for this group of compounds, the antihypertensive action was not linked to a single specific mechanism but to the convergence of several minor effects. Indeed, the inhibition of the activation of adrenoreceptor was observed in vitro, but not for all compounds in mice, and only variable degrees of calcium antagonism were observed in guinea pig (g.p.) ileum and atria but not in the rat portal vein [28]. Somewhat intriguing is the simultaneous high anti-inflammatory and antihypertensive activity of compound **86**.

In addition to some minor pharmacological effects, it is interesting to mention the observation of antihypercholesteremic activity for compound **84** at a dose of 300 mg/kg p. os, as it was already observed for compounds **57** and **62**. Collateral hypocholesterolemic activity was previously reported for some butyrophenone antipsychotics such as haloperidol and trifluperidol that, indeed, inhibit several enzymes involved in cholesterol biosynthesis (Δ^7^-reductase, Δ^8^⁻^7^-isomerase, and Δ^14^-reductase, in order of decreasing participation in biosynthesis) [32,33].

It is known that Δ^8^⁻^8^-isomerase is related to the sigma-1 (σ_1_) receptor protein (66% homology) [34] and that the haloperidol, besides having affinity to D_2_ receptor, which is mainly responsible for its antipsychotic activity, exhibits also high affinity to sigma-1 receptor (Ki = 6 nM). Ligands to sigma-1 receptor, devoid of affinity to D_2_ receptor, could be worthy of investigation as potential cholesterol-lowering agents to be eventually associated with the well-established statins (acting as HMG-CoA reductase inhibitors).

Indeed, in a preliminary general screening of prototypic compound **83** (very close to **84**), a high affinity to σ_1_ receptor (Ki = 12 nM) was observed, rather close to that of haloperidol.

## 5. Affinity to Sigma Receptors

**Figure 9:**
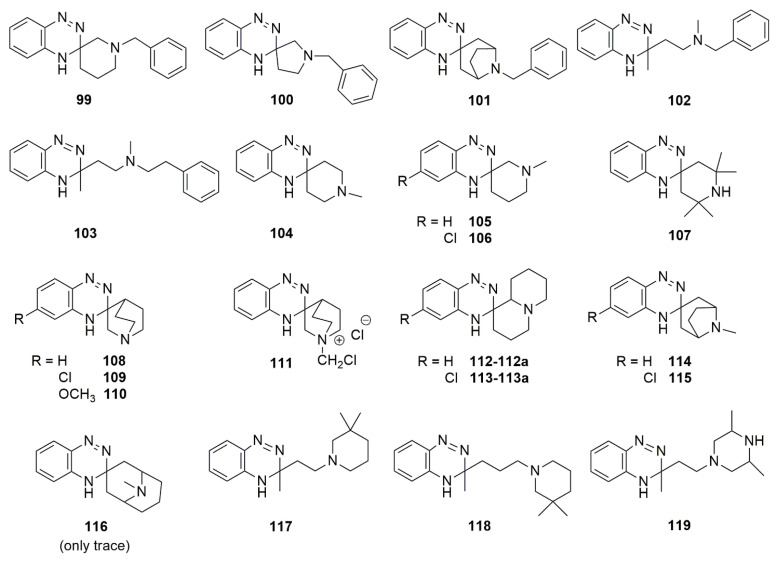
Further synthesized basic 3,3-disubstituted 3,4-dihydro-1,2,4-benzotriazines.

Since the affinity for sigma receptors can have an important role in many pharmacological fields, several 3,4-dihydro-1,2,4-benzotriazine derivatives, (either related to the above spiropiperidines or to other kinds of amino compounds) were synthesized [11,28,35,36] for testing for affinity to such receptors. From this perspective, compounds related to Figure 7 and Figure 9 could be differently assembled in two subsets, containing (**69**–**103**) or not (**104**–**119**) an aryl moiety in the side chain.

Sigma receptors, formerly considered a subclass of opioid receptors occur in at least two types of binding sites, called σ_1_ and σ_2_. They are distributed in the CNS and in several peripheral tissues, and they are also overexpressed in many kinds of tumor cells. The specific functional roles of the two subtypes have been progressively defined, acquiring clinical relevance [37,38]. By themselves, or in concerted mechanisms involving other receptors, sigma receptors participate in motor function, regulation of smooth muscle contraction, neurotransmitter synthesis and release, opioid analgesia modulation, neuro-degeneration and cognitive impairment, immune homeostasis, and sterol biosynthesis.

The achievement of atypical antipsychotics, with dual affinity to σ and 5-HT_2_ receptors, and of agents improving cognitive deficits have been particularly pursued [39].

Additionally, sigma receptors play a role in tumor cell growth and proliferation. Particularly, the expression of σ_2_ receptor reflects the proliferative status of cells, being 10-fold higher in proliferating than in quiescent ones. It is worth noting that both sigma-1 antagonists and sigma-2 agonist can display anticancer action [40,41].

Many structurally disparate compounds are able to bind sigma receptors, but only a few bind with high affinity and selectivity to each single subtype. Among the latter, some spiro-piperidine derivatives present a particular interest either for the high level of affinity and selectivity or for the association of the affinity for σ_1_ to that for other specific receptors preluding to antipsychotic, analgesic, nootropic, or antitumor activities. Examples (**120**–**127**) are found in Table 1.

At the present time, only some of the prepared compounds included in Figure 7 and Figure 9 have been tested for affinity to sigma receptors, and the results have been collected in Table 2 [35]. Very importantly, all the tested compounds bearing a phenyl nucleus in the basic side chain display nanomolar affinity for the σ_1_ receptor subtype, and it is evident, at first glance, that there is some structural analogy between them and the tetraline- and benzopyrane/thiopyrane-derived ligands in Table 1.

Even though only a small number of compounds have been tested for binding to the σ_2_ receptor, it is evident that the affinity to this receptor is lower than that to σ_1_, with a selectivity rate ranging from 11 to 277. It is worth noting that for compound **69**, other Ki values have been observed for both sigma subtypes, particularly for sigma-2, when the usual label [^3^H]-DTG is replaced by the more specific [^3^H]-ifenprodil, and the selectivity index can reach a value of 7033.

Therefore, whatever values are taken into consideration, compound **69** can be considered one of the most potent and selective ligands for σ_1_ receptor; moreover, this compound is characterized by very poor affinity versus D_2_ and 5-HT_2_ receptors (Ki for the latter = 1.47 µM). Commonly, spiro[1,2,4-benzotriazine-3(4H),4′-(1′-substituted piperidines)] (**69**–**78**, **83**, **86**, **99** and **100**) display higher affinity than the remaining (non-spiro) compounds. The Ki values of the 19 tested compounds (Table 2) can be grouped into two clusters: nine compounds in the range of 0.6–4 nM, eight in the range of 11–47 nM, and only two with Ki ≥ 110 nM.

The lengthening of the aliphatic linker, between the piperidine nitrogen and the terminal aryl moiety, from one to four methylenes does not significantly change the affinity, which is, however, improved when five methylenes are present. Affinity versus the σ_2_ receptor increases with the increase in the chain length. The presence of a para substituent on the N-benzyl residue produces only small variations in the Ki value, which, anyhow, is lowest for the chloroderivative **71** (1.25 nM) and highest for the methoxy one **73** (4 nM).

A comparison of the butyrophenone derivatives **78**, **83**, and **86** with the phenylbutyl derivative **76** suggests that the carbonyl group is not useful for the affinity to the receptor.

When the link of the dihydrobenzotriazine ring to piperidine is moved from C-4 to C-3 (as in compounds **69** and **99**), a decrease in affinity is observed (2.8 nM → 10.6 nM). However, the real effect of this structural modification cannot be correctly evaluated without the separation and testing of the enantiomers composing the racemate **99**. Similar considerations may hold for the comparison of compound **69** to compound **100**, which also is a racemate. Nevertheless, by comparing the two racemates **99** and **100**, it is evident that the smaller pyrrolidine ring is less qualified than the piperidine ring to fit the σ_1_ receptor subtype.

On the other hand, it is observed that both the N-benzyl-piperidine derivative **69** and the more cumbersome N-benzyl-nortropane derivative **101** exhibit the same Ki value (2.8 and 2.6 nM, respectively), suggesting that some bulkiness around the basic nitrogen is tolerated by the receptor, provided that the benzyl residue can maintain a proper spatial disposition. This result supports further the existence, as claimed by Glennon [47,48], of a bulk tolerating region in the σ_1_ receptor.

**Figure 10:**
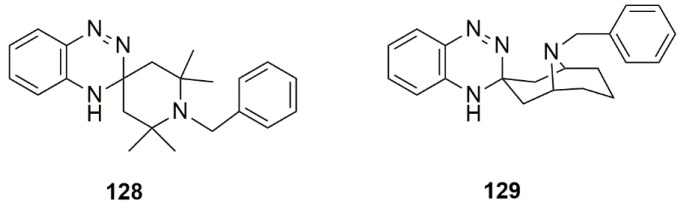
Unobtained highly bulky 3,4-dihydro-1,2,4-benzotriazine derivatives.

To support further this observation, the synthesis of compounds characterized by a still higher bulkiness around the basic nitrogen, such as compounds **128** and **129** (Figure 10), was attempted but failed.

Indeed, the required N-benzyl-2,2,6,6-tetramethyl-piperidin-4-one prepared as indicated by Guareschi [49] is not a piperidine derivative but an open isomer, as successfully demonstrated by Banert et al. [50]. Therefore, when treated with 2-nitrophenylhydrazine, a C_13_H_14_N_4_O compound is formed, corresponding to the salt between benzylamine and 1-hydroxybenzotriazole [35].

It is worth noting that starting from 2,2,6,6-tetramethylpiperidone, it is possible to obtain the corresponding 2-nitrophenylhydrazone, and from this, the dihydrobenzotriazine **107**. The latter results suggest reattempting the preparation of **128**, starting from the authentic N-benzyl-2,2,6,6-tetramethyl-piperidin-4-one prepared according to the new method of Banert et al. [50].

**Scheme 10:**
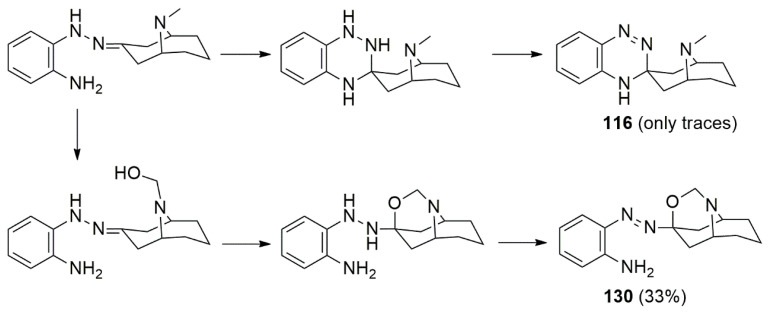
Alternative ring closure of 2-amino-phenylhydrazone of N-methylgranatanone.

On the other hand, the failure of the synthesis of compound **129** is probably due to the supposed higher steric hindrance inherent to the granatanone ring even in comparison to the tropanone, whose corresponding dihydrobenzotriazine derivative **101** was formed with only a 14% yield. When the steric hindrance slows down the formation of the expected dihydrobenzotriazine **116**, other kinds of intramolecular reactions may take place, as a slow oxidation of the N-methyl group of N-methylgranatanone (pseudo-pelletierine) to N-hydroxymethyl, which, in turn, may compete favorably with the amino group for the addition to the hydrazone double bond (Scheme 10).

The structure of the obtained azo compound **130** is soundly supported by UV ^1^H-NMR, ^13^C-NMR and DEPT spectra, while the sequence leading to its formation is just one of those that could be envisaged [36].

The spiro[1,2,4-benzotriazine-3(4H),4′-(1′-methyl)piperidine] **104** exhibited an affinity for the σ_1_ receptor that was three orders of magnitude lower (Ki = 3410 nM) than the corresponding benzyl derivative **69**, thus underlining the importance of an aromatic moiety at the end of the side chain.

**Figure 11:**
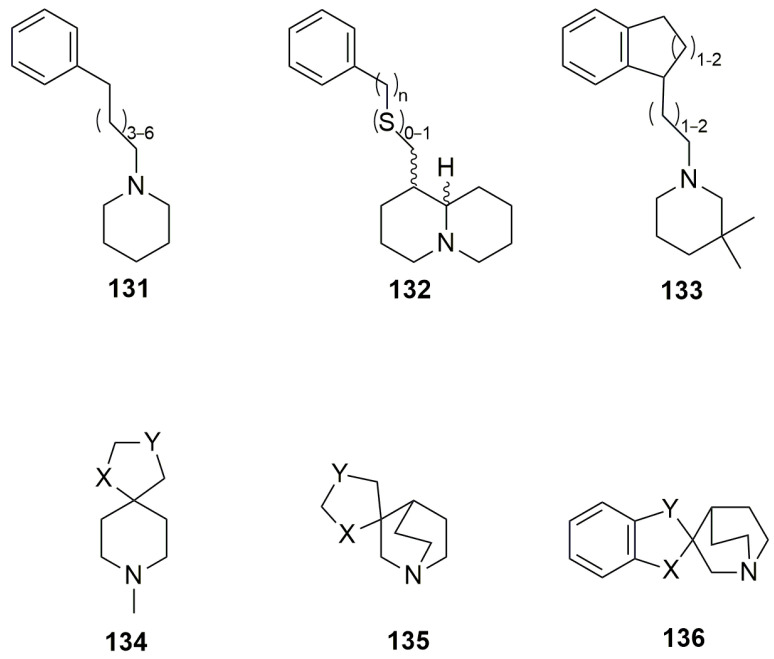
General structures of active compounds devoid of a second aromatic ring.

However, several kinds of potent ligands for σ_1_ receptor are known that are devoid of a terminal aromatic ring, provided that the basic nitrogen is joined to the aromatic head through a long linker (**131** [47,51]) or is embodied in a bulky moiety, like the bicyclic quinolizidine (**132** [52,53]) or the 3,3-dimethylpiperidine (**133** [54]). Moreover, several kinds of spiro derivatives of N-methylpiperidine (**134**) or, even better, of 3-spiro derivatives of quinuclidine (**135**, **136**) have been shown to exhibit central muscarinic activity or an agonistic effect on the α_7_ nicotinic receptor [55,56,57,58], which is useful in cognitive enhancement (Figure 11).

Thus, the dihydrobenzotriazines devoid of a second aromatic moiety **104**–**119** (Figure 9) were synthesized in order to verify the possibility to still bind to sigma receptors or to display other activities such as on the central cholinergic system [36].

However, to date, no further data on the affinity to sigma receptors have been achieved. Instead, some data have been obtained regarding the affinity to muscarinic and nicotinic receptors, concerning compounds **105**–**108**, **112**, and **114**. Compounds **108** and **114** exhibit an IC_50_ value of 2 µM and 4.7 µM, respectively, for the displacement of [^3^H] QNB from muscarinic rat cortex receptors. Even poorer is the affinity to central nicotinic binding sites; the best compound (**105**) displaced [^3^H]cytisine by only 44% at a 10 µM concentration [36].

A final observation concerns the different pharmacological behaviors of some derivatives of 4-oxo-spiro[benzopyran-2,4′-piperidine]; the N-benzyl derivative **124** is a potent ligand for the σ_1_ receptor (see Table 1) [43], while the N-(napht-1-yl)ethyl derivative is a potent ligand for α_1_ adrenergic receptors, whose further selectivity versus the α_1A_ subtype (important to have an agent for the treatment of BPH) can be improved by particular substitution on the benzopyrane moiety [59].

It would be interesting to verify if the introduction of the (napht-1-yl)ethyl residue and of suitable substituents on the spiro (3,4-dihydro-1,3,4-benzotriazine)-3,4′-piperidine scaffold could produce an upsurge in the affinity to the α_1A_ receptor. To that end, compounds **88**–**90** and **96**–**98** (Figure 7) have been synthesized, but no biological results are at present available.

## 6. Cytotoxicity and Antiproliferative Activity

Regarding the role played by sigma receptors (σ_1_ antagonism and σ_2_ agonism) in cell proliferation [37,38,60], compounds **69** and **76**–**78** have been tested [61] in two human breast cancer cell lines, namely, MCF-7 and MDA-MB231, which are estrogen-responsive and estrogen-unresponsive, respectively (Table 3).

Both the affinity to sigma receptors and the antiproliferative activity increased with the increasing length of the side chain, although the presence of the carbonyl group (**78**) was somewhat detrimental for both. The absence of σ_1_ receptors in the human MCF-7 cell line has been indicated [62], and this could explain the lower sensitivity towards the tested compounds in comparison to MDA-MB231 cells, for which both σ_1_ and σ_2_ binding sites may contribute to growth inhibition. After 48 h of treatment, morphological alteration progressed from loss of continuity of the monolayer to an increasing number of round cells, cell detachment, and the presence of falciform-like dead cells and cell debris, which was consistent with an apoptotic process. These observations agree with the results of other authors, demonstrating the ability of certain σ_2_ receptor agonists to induce cell death by apoptosis in breast tumor cell lines [63].

The benzopyranone derivative **124**, which displayed very high affinity to the σ_1_ receptor (Table 1), did not inhibit the growth of MCF-7 cells, while it was active (IC_50_ = 10 µM) against MCF-7/ADR cells highly expressing the σ_1_ receptor [43].

It is worth noting the cytotoxic effect on MT-4 cells (modified human T-cells) that is produced by the introduction of substituents on the aromatic rings of compound **83** (IC_50_ = 73 µM). The introduction, at position 6, of substituents, either electron-withdrawing such as CF_3_ (**85**) or electron-releasing such as OCH_3_ (**86**), clearly enhances cytotoxicity with IC_50_ = 33 µM and 15 µM, respectively. On the other hand, the elimination of fluorine from the terminal phenyl nucleus of **86** strongly reduces the cytotoxicity (**86** → **79**; IC_50_ from 15 µM to >200 µM). The application of these observations to the compounds presented in Table 3 might, eventually, improve their antiproliferative activity on breast cancer cells.

Finally, compound **86** was examined for antiproliferative activity within the “National Cancer Institute Developmental Therapeutics Program” (Bethesda, Rockville, MA, U.S.A.) against a panel of 60 human cancer cell lines and exhibited IC_50_ (GI_50_) values for all of them in the range from 12 to 40 µM (Report date: 14 May 1998; NSC 703163-Q(1)). Particularly sensitive (GI_50_ < 20 µM) were leukemia, colon cancer, melanoma, and breast cancer cell lines.

Even if many of the synthesized compounds remain to be tested, the described results clearly indicate that 3,4-dihydro-1,2,4-benzotriazine derivatives bearing at position 3 a basic arylalkyl chain are valuable (often nanomolar) ligands for the sigma receptor (Table 2) and, therefore, are suitable starting points for developing neurotropic as well as antiproliferative agents.

## 7. Dihydrobenzotriazine Derivatives as Prodrugs: Miscellanea

As illustrated in the section “Chemical properties”, the 3,4-dihydro-1,2,4-benzotriazines can give rise, under mild operating conditions, to starting ketones and aromatic amines, and it is reasonable that such reactions may take place, more or less rapidly, also in vivo, as an effect of azoreductases [14,15] and/or various hydrolytic enzymes. Therefore, the relevant compounds could display their biological activities as such or through the degradation products if these are biologically active by themselves. In other words, they could act as prodrugs.

Moreover, in the case of biologically active but highly lipophilic ketones, (such as the steroidal hormones) it is possible to enhance the hydrophilicity by building up (on their C-3 position) a dihydrobenzo/pyridotriazine nucleus bearing a basic or acidic function to be salified.

Vice versa, in the case of aryl/heteroaryl amines that are biologically active but excessively hydrophilic, a dihydrobenzotriazine nucleus could be constructed using a lipophilic but biologically inert ketone.

**Figure 12:**
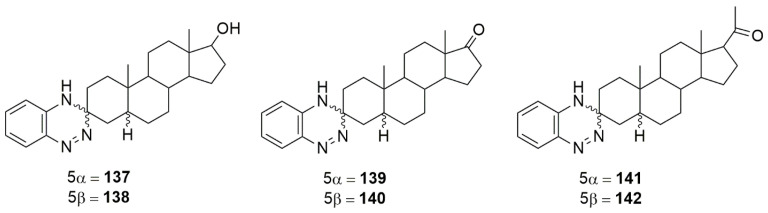
Steroidal 3,4-dihydro-1,2,4-benzotriazine derivatives with hormonal activity.

Starting from 5α- and 5β-dihydrotestosterone, 5α- and 5β-androstan-3,17-diones, and 5α- and 5β-pregnan-3,20-diones, the corresponding dihydrobenzotriazine derivatives **137**–**142** were obtained [8] (Figure 12).

The compounds **137**–**138** (from 5α/5β-dihydrotestosterone) and compounds **139**–**140** (from 5α/5β-androstan-3,17-dione) were tested for androgenic activity using the test of male frog thumb callosity and of mouse seminal vesicle. In the former assay, 5α and 5β derivatives were active and inactive, respectively, as were the corresponding ketones. However, in the latter assay, the 5β-isomer **138** was active, even though the 5β-ketone was inactive. Therefore, this spiro dihydrobenzotriazine-steroid itself must be active, independently from the release of the ketosteroid; thus, the heterocyclic portion of the molecule may fulfil quite more than a latentiating function.

This result promotes the realization of synthetic hormones and eventually antihormones by synthesizing additionally substituted dihydrobenzotriazines or 3,4-dihydro-pyrido-1,2,4-triazines.

These results underline the importance that can be assumed of 3,4-dihydro-1,2,4-benzotriazine **141**–**142** and the corresponding 3,4-dihydro-1,2,4-pyridotriazine derivatives in consideration that 5α-dihydroprogesterone, together with other neurosteroids, act as neuro-regulating factors [64,65,66,67] in neuro-psychiatric disorders.

Lastly, an attempt to modulate the hydrophilic/lipophilic balance of biologically active amino derivatives of pyridine, such as 4-aminopyridine (4-AP; fampridine) and 3,4-diaminopyridine (3,4-DAP, amifampridine), is described. These amines represent approved drugs for the symptomatic treatment of myasthenic symptoms (LEMS), multiple sclerosis (MS), and botulinum intoxication [68,69,70]. 3,4-Diaminopyridine is many folds more potent than 4-AP as a K^+^ channel blocker and less toxic because of its slower passage across the BBB; however, it presents some shortcomings due to its higher hydrophilicity.

**Scheme 11:**
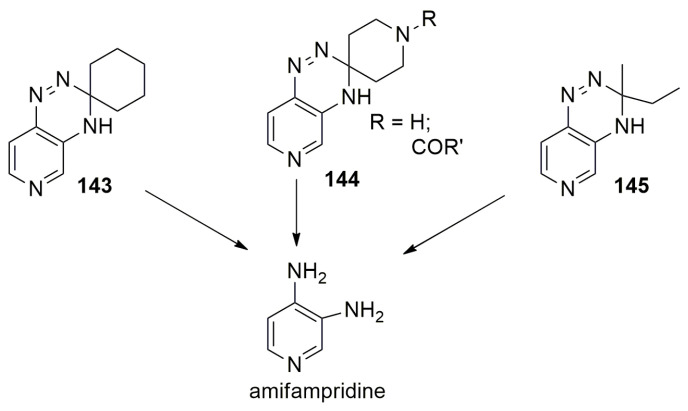
Possible prodrugs of amifampridine.

To overcome this issue, some possibly extended-release forms of 3,4-DAP have been synthesized as compounds **143**–**145** [71,72]. These compounds, through the intervention of azo-reductases, can release the active 3,4-diaminopyridine with different graduality (Scheme 11).

No biological tests have been carried out, so far, on these compounds.

## 8. Conclusions and Perspectives

The 1,2,4-benzotriazine nucleus was firstly synthesized in 1889 by Bischler, and since then a great number of derivatives have been prepared and investigated for different biological activities [7,12], and some of them have reached clinical trials for the treatment of certain cancers and macular degeneration. From this fundamental aromatic nucleus, different kinds of dihydro derivatives can be formally expected. The loss of planarity of the bicyclic system allows hitting further cellular targets and displaying additional activities.

Derivatives of 1,4-dihydro-1,2,4-benzotriazine were described in the 1950s, followed in 1968 by the strictly related 1,4-dihydro-1,2,4-benzotriazin-4-yl radicals (Blatter radicals) that progressively assumed a fundamental relevance in the field of organic electronics [73,74]. Vicinal dihydro derivatives of 1,2,4-benzotriazine were never described until 1969 when the structure of cyclohexane-3-spiro-3,4-dihydro-1,2,4-benzotriazine [3] was attributed by F. Sparatore and Cerri to a compound obtained by Perkin and Riley in 1923. From that time on, many 3,3-disubstituted 3,4-dihydro-1,2,4-benzotriazines have been synthesized by a single research group and assayed for several pharmacological activities. The most diffused were antihypertensive, anti-inflammatory and diuretic activities, among which emerged compounds **86**, **67**, and **68** as antihypertensive; **86** and **39** as anti-inflammatory, and **42** as diuretic. Less diffuse were analgesic (**83**), platelet antiaggregating (**84**), trachea relaxant (**57**), and anti-convulsant (**27**) activities. A very weak reduction in serum cholesterol was observed for a few compounds (**57**, **62**, **84**), not structurally correlated to any known hypocholesterolaemic agent. Therefore, these compounds deserve further investigation.

3,3-Disubstituted 3,4-dihydro-1,2,4-benzotriazines represent a novel class of sigma receptor ligands, with nanomolar affinity and which, among other possibilities, could promote the development of interesting antitumor agents, as seen for compound **86**.

Moreover, it has been shown that the construction of a 3,4-dihydro-1,2,4-benzotriazine nucleus on the C-3 position of steroidal hormones could latentiate and even improve the hormonal activity; thus, modified 5α-dihydroprogesterone (which is the endogenous ligand for the σ_1_ receptor), as **141** and **142**, could be of interest in the field of neuro-degenerative diseases.

As has been often pointed out, some of the synthesized compounds have not yet been evaluated for activity and may represent opportunities for further investigation.

Therefore, resuming the interrupted study of 3,3-disubstituted 3,4-dihydro-1,2,4-benzotriazines (and of their isosteric pyridine analogs) would fulfil the potential of this promising, little exploited class of compounds.

## Figures and Tables

**Table 1:** Affinity to sigma receptors of some 4-spiro-(1-benzyl)piperidines.

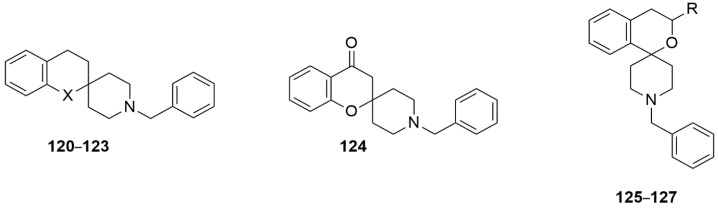					
Compounds		K_i_ (nM)		Ratio	References
		σ_1_	σ_2_	σ_2_/σ_1_	
**120** (spipethiane)	X = S	0.50	416	832	[42]
**121**	O	0.62	22	35.5	[42]
**122**	CH_2_	0.44	14	31.8	[43]
**123**	C=O	1.40	10	7.14	[42,44]
**124**		0.21	302	1440	[43]
**125**	R = H	0.69	99.7	146	[45]
**126**	OCH_3_	1.20	3500	2710	[45]
**127**	C≡N	1.54	1590	1030	[46]

**Table 2:** Affinity to sigma receptors of representative 3,3-disubstituted 3,4-dihydro-1,2,4-benzotriazines.

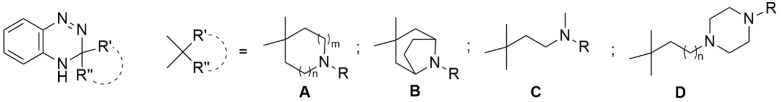									
Compounds		Structure	m	n	R	Ki (nM)			Ratio
						σ_1_^a^	σ_2_^b^		σ_2_/σ_2_
**69**		A	1	1	CH_2_-C_6_H_5_	2.8 0.6 ^c^	640 4220	^cd^	229 7033
**70**		A	1	1	CH_2_-C_6_H_4_-F(4)	2.8			
**71**		A	1	1	CH_2_-C_6_H_4_-Cl(4)	1.25	346		277
**72**	^e^	A	1	1	CH_2_-C_6_H_4_-CH_3_(4)	2.5			
**73**		A	1	1	CH_2_-C_6_H_4_-OCH_3_(4)	4.0			
**74**		A	1	1	CH_2_-CH_2_-C_6_H_5_	4.0 ^c^			
**76**	^e^	A	1	1	(CH_2_)_4_-C_6_H_5_	3.8	43		11.3
**77**	^e^	A	1	1	(CH_2_)_5_-C_6_H_5_	0.96	52		55.3
**78**	^e^	A	1	1	(CH_2_)_3_-CO-C_6_H_5_	14.0	277		19.8
**83**		A	1	1	(CH_2_)_3_-CO-C_6_H_4_-F(4)	12.0 ^c^			
**86**		A ^f^	1	1	(CH_2_)_3_-CO-C_6_H_4_-F(4)	10.8	656		60.7
**99**		A	0	2	CH_2_-C_6_H_5_	10.6			
**100**		A	0	1	CH_2_-C_6_H_5_	47.0			
**101**		B			CH_2_-C_6_H_5_	2.6			
**102**	^e^	C			CH_2_-C_6_H_5_	35.0			
**103**	^e^	C			CH_2_-CH_2_-C_6_H_5_	37.5			
**104**		A	1	1	CH_3_	3410			
**57**		D		1	C_6_H_5_	110.0			
**65**		D		2	C_6_H_5_	25.0			
**haloperidol**						6.0	59		9.8
**haloperidol**	^c^					2.0			

^a^ Displacement of [^3^H](+)pentazocine. ^b^ Displacement of [^3^H]DTG (unless otherwise stated). ^c^ Results of MDS-Panlabs Pharmacology Service, Bothell, WA, USA. ^d^ Displacement of [^3^H]ifenprodil. ^e^ Tested as hydrochloride. ^f^ 6-OCH_3_ derivative.

**Table 3:** Affinity to sigma receptors and antiproliferative activity against two breast cancer cell lines.

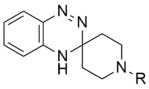					
Compounds	R	K_i_ (nM)		IC_50_ (µM)	
		σ_1_	σ_2_	MCF-7	MDA-B231
**69**	CH_2_-C_6_H_5_	2.8	640	340	148
**76**	(CH_2_)_4_-C_6_H_5_	3.8	43	83	36
**77**	(CH_2_)_5_-C_6_H_5_	0.9	52	90	33
**78**	(CH_2_)_3_-CO-C_6_H_5_	14.0	277	169	85

## Data Availability

Not applicable.
